# Influences of Fermentation Temperature on Volatile and Non-Volatile Compound Formation in Dark Tea: Mechanistic Insights Using *Aspergillus niger* as a Model Organism

**DOI:** 10.3390/foods15030441

**Published:** 2026-01-26

**Authors:** Rida Niaz, Mingjin Li, Qian Pu, Anlan Qu, Tianci Shen, Minghui Qi, Chengtao Wang, Lixia Chen, Shuang Wu, Youyi Huang

**Affiliations:** National Key Laboratory for Germplasm Innovation & Utilization of Horticultural Crops, Tea Science Department of College of Horticulture and Forestry of Huazhong Agricultural University, Wuhan 430070, China; ridaniazgcuf@gmail.com (R.N.); limingjin0511@163.com (M.L.); puqian_430@webmail.hzau.edu.cn (Q.P.); qal010905@126.com (A.Q.); 18749083856@163.com (T.S.); qmh19115429@163.com (M.Q.); 15083454718@163.com (C.W.); 15827476848@163.com (L.C.); wushuang522@aliyun.com (S.W.)

**Keywords:** dark tea, variable temperature fermentation, *Aspergillus niger*, characteristic compounds, metabolomics

## Abstract

The mechanism of the quality formation of dark tea is not fully clear, particularly under variable fermentation temperatures. In this study, the tea fermented with *Aspergillus niger* (AN) at 25 (AN25) and 37 °C (AN37) exhibited the highest quality. Different fermentation temperatures primarily influenced the degradation of fatty acids and the hydrolysis of glycosides in the tea, with 37 °C being the most favorable for the release and accumulation of volatile compounds. Eighteen key volatiles were identified. Among these, benzaldehyde (a 120.9% increase compared to CK), α-ionone (957.8%), linalool (172.2%), and nonanal (22.8%) were present at high levels in AN37, and these compounds served as the main aroma contributors. Inoculation with AN and fermentation temperature primarily influences the levels of total polyphenols, organic acids and their derivatives, as well as amino acids and their metabolites in dark tea. Total polyphenols, flavonoids, and nucleotide and its metabolites were more rapidly consumed at 25–37 °C, contributing to the improved taste of the tea infusion. Additionally, EGC, GC, melezitose, and sucrose showed significant negative correlations with the taste quality of the tea infusion (*p* < 0.05). These results are conducive to further understanding of the quality formation of dark tea.

## 1. Introduction

Tea is one of the most popular beverages in the world. Depending on the processing technology and the quality, tea can be categorized into black tea, green tea, oolong tea, yellow tea, white tea, and dark tea. Among them, dark tea, a uniquely post-fermented tea in China, is primarily characterized by a distinctive aged flavor and woody aroma formed by microbial fermentation [1]. Due to differences in raw leaf materials and processing technologies, dark tea mainly includes Hubei Qingzhuan tea, Yunnan Pu-erh tea, Sichuan Tietban tea, Hunan dark tea, and Guangxi Liupao tea [2]. In the past decade, dark tea has gained widespread popularity due to its unique flavor and health benefits [3].

The unique flavor and health benefits of dark tea stem from a unique process of pile fermentation. Pile fermentation is a critical process in forming the characteristic qualities of dark tea, characterized by the growth and succession of microbial communities [4]. During the pile fermentation of dark tea, there is a distinct rise and fall of fermentation temperature, which not only affects the succession of microbial communities but also influences various enzyme activities [5], thereby impacting the transformation of quality components in tea leaves. Currently, it is believed that the mechanisms of pile fermentation involve microbial fermentation, enzymatic oxidation, and hydrothermal reactions, all of which originate from the growth and metabolism of microorganisms [1,5]. During pile fermentation, microorganisms secrete extracellular enzymes, promoting the degradation of proteins, pectin, and cellulose, as well as the enzymatic oxidation of catechins [4]. Additionally, an appropriate temperature during pile fermentation is crucial for maintaining microbial growth and high enzymatic catalytic reactions that hydrolyze aroma precursors and produce rich aromas [1]. However, the mechanism of the quality formation of dark tea is still not fully clear, particularly the effects of different temperatures on pile fermentation [5].

There are many kinds of microbes in the pile fermentation of dark tea, but *Aspergillus niger* (AN) has been identified as the dominant filamentous fungus, with the highest isolation frequency [5]. AN can produce multiple extracellular enzymes, accelerating the transformation of flavor substances [6]. To reveal the effect of fermentation temperature on sensory quality and quality components of dark tea, dark tea was inoculated with AN and fermented at 25 °C, 30 °C, 37 °C, 40 °C, and 45 °C, and the metabolites were analyzed by gas chromatography–mass spectrometry (GC-MS) and liquid chromatography–mass spectrometry (LC-MS). This approach facilitates a deeper understanding of the quality formation of dark tea.

## 2. Materials and Methods

### 2.1. Materials and Reagents

The sun-dried green tea was processed at the Fangshan’an Tea Factory (Chibi City, Hubei Province, China). The raw material consisted of one bud and four leaves plucked from the *Camellia sinensis* cv. Chibi Group cultivar. AN was isolated from Qingzhuan tea, meets the safety standards specified in the Chinese National Standard GB/T 2761-2021 [7], and is preserved in our laboratory at the Tea Department of Huazhong Agricultural University (Wuhan, China). For GC–MS analysis, ethyl decanoate (Shanghai, China) served as the internal standard, with C_7_-C_40_ n-alkanes (Shanghai Yuanye, China) used for the calculation of linear retention indices (RI). Chromatographically pure methanol, ethanol, and acetonitrile were supplied by Darmstadt, Germany, and Waltham, MA, USA.

### 2.2. Tea Samples

The spore suspension of AN was prepared according to the method reported by [8]. AN was cultured on Potato Dextrose Agar (PDA) plates at 37 °C for approximately 10 h, and the final spore concentration was adjusted to 1.0 × 10^7^ spores/mL. Each 100 mL bottle was filled with 20 g of sun-dried green tea leaves and sterilized at 121 °C for 15 min using an autoclave (MZQ-JDMD-0.6 Wuhan Jianghan Medical Pharmaceutical Equipment, Wuhan, China). After cooling, a 3 mL AN spore suspension was inoculated into the bottles along with 7 mL of sterilized ddH_2_O to adjust the tea moisture content to 40% [8]. The bottles were then incubated at 25 °C (AN25), 30 °C (AN30), 37 °C (AN37), 40 °C (AN40), and 45 °C (AN45) for 5 d. 37 °C is typically the optimal temperature for the growth of AN [6,9], so it was used as a reference temperature point for comparison. For the control group (CK), sterilized sun-dried green tea leaves were moistened with 10 mL of sterilized water and incubated at 30 °C for 5 d. After 5 d, the various fermented tea samples were dried at 40 °C for 4 h. Portions of the dried samples were ground into powder, and all samples were stored at −80 °C for further analysis. All treatments were performed with three independent biological replicates.

### 2.3. Sensory Evaluation

According to the Chinese National Standard (GB/T 23776-2018) [10], sensory evaluations were conducted on each tea sample. Briefly, 3.0 g of tea was accurately weighed into a 150 mL cylindrical tasting cup. Then, 150 mL of boiling water was added, and the mixture was steeped for 2 min before the tea infusion was filtered. The infusion color, aroma, and taste were evaluated. Subsequently, a second brewing was carried out by steeping the same leaves for 5 min, and the resulting infusion was filtered and sequentially evaluated for color, aroma, taste, and residue leaves. The evaluation primarily focused on the second infusion, with the first infusion serving as supplementary reference. The evaluation panel consisted of five professionally trained assessors (three female and two male, aged 25–35 years) from Huazhong Agricultural University. All panelists hold advanced tea sensory evaluation certificates and have more than five years of experience in tea sensory evaluation. During the evaluation process, each panelist worked independently without communication. Each sample was evaluated in three independent sessions conducted on separate days to account for intra-panelist variation and daily sensitivity fluctuations. The total score was calculated based on the following weightings: infusion color (15%), aroma (35%), taste (40%), and residue leaves (10%). The final sensory score for each sample was determined as the mean value across the three replicate sessions. The variability among sessions was reported as the standard deviation.

### 2.4. Determination of Major Quality Compounds

The contents of water extracts (WEs), tea polyphenols (TPs), free amino acids (FAAs), and tea pigments were determined according to the Chinese National Standards GB/T 8305-2013 [11], GB/T 8313-2018 [12], and GB/T 8314-2013 [13], respectively. The content of soluble sugars (SS) was measured using an ultraviolet spectrophotometer [14].

### 2.5. Determination of Catechins, Gallic Acid, Caffeine

Tea powder (0.2 g) was mixed with 10 mL of 70% methanol in a 10 mL centrifuge tube. The mixture was vortexed, then allowed to stand at 4 °C for 15 h. After centrifugation at 11,000× *g* for 5 min, the supernatant was filtered through a 0.45-μm membrane for analysis. Compound detection was performed using an Agilent 1200 series HPLC system (Agilent, CA, USA) equipped with an Agilent TC-C_18_ column (250 × 4.6 mm, 5 μm; Agilent, CA, USA). The detection wavelength was set at 278 nm. The remaining HPLC parameters were as described by Pu et al. [14].

### 2.6. Detection of the Volatile Compounds

Accurately weighed tea powder (1.00 g) was placed in a 20 mL headspace vial. Then, 5 mL of boiling water and 10 μL of a 10.67 μg/mL ethyl decanoate internal standard solution (dissolved in dichloromethane) were added. The headspace vial was placed in a 60 °C water bath for extraction. A sampling handle (57357U, Supelco) equipped with a 75 μm CAR/PDMS solid-phase microextraction (SPME) fiber (Supelco, Bellefonte, PA, USA) was inserted into the vial. After equilibrating for 10 min, the fiber was exposed to the headspace for 30 min for analyte adsorption. Upon completion, the SPME fiber was retracted and immediately inserted into the GC-MS injection port for 5 min of thermal desorption.

Volatile compounds were analyzed using a TRACE DSQ II GC-MS system (Thermo Fisher Scientific, Waltham, MA, USA) equipped with a DB-5MS capillary column (30 m × 0.25 mm × 0.22 μm; Agilent, Santa Clara, CA, USA). Helium (purity > 99.999%) served as the carrier gas at a flow rate of 0.8 mL/min. The oven temperature was initially set to 50 °C and maintained for 8 min, then raised to 90 °C at a rate of 10 °C/min and sustained for 2 min, subsequently raised to 180 °C at a rate of 2.5 °C/min and sustained for 2 min, and finally raised to 300 °C at a rate of 20 °C/min and sustained for 2 min. The mass spectrometer was operated in electron ionization (EI) mode at 230 °C. Data were acquired in full-scan mode over a mass range of 30–650 *m*/*z*, with an electron energy of 70 eV. The identification of volatile compounds was performed by first matching their mass spectra with the National Institute of Standards and Technology (NIST) database (with a match factor > 700), followed by comparing the calculated retention indices (RIs, n-alkanes C_7_-C_40_) with theoretical RIs (±20). Volatile compounds meeting both the match factor and RI criteria were considered as the identification results. Quantification was performed using the internal standard (ethyl decanoate) method according to Fang et al. [15].

### 2.7. Calculation of Odor Activity Value

The calculation of odor activity value (OAV) referenced a previous study [15].

### 2.8. Detection of the Non-Volatile Compounds

Non-volatile compounds in fermented tea leaves were extracted by adding 400 μL solution (Methanol: Water = 7:3, *V*/*V*) containing internal standard to 20 mg of the sample, followed by vortexing for 3 min. The mixture was subsequently sonicated in an ice bath for 10 min, vortexed for 1 min, and then incubated at −20 °C for 30 min. After centrifugation at 11,000× *g* for 10 min (4 °C), the sediment was discarded, and the supernatant was centrifuged again at 11,000× *g* for 3 min (4 °C). A 200-μL aliquot of the resulting supernatant was transferred for LC-MS analysis. The analysis was performed using an ultra-high-performance liquid chromatography system (LC-30A, Shimadzu, Kyoto, Japan) coupled with a TripleTOF 6600+ mass spectrometer (SCIEX, Framingham, MA, USA). Separation was achieved on a Waters ACQUITY Premier HSS T_3_ Column (1.8 µm, 2.1 mm × 100 mm). The mobile phase consisted of 0.1% (*v*/*v*) formic acid in water (solvent A) and 0.1% (*v*/*v*) formic acid in acetonitrile (solvent B). The remaining LC-MS parameters were as described by [15]. The detected metabolites were submitted to Kyoto Encyclopedia of Genes and Genomes (KEGG) and Human Metabolome Database (HMDB) databases for qualitative comparison and annotation in the I-Sanger cloud platform (Majorbio, Shanghai, China). For quality control (QC), a pooled sample was prepared by mixing equal volumes of all samples to assess instrument stability and repeatability.

### 2.9. Statistical Analysis

Each tea sample was analyzed in triplicate. A one-way ANOVA followed by Tukey’s post hoc test was performed using SPSS (Version 20.0). Principal component analysis (PCA), partial least squares discriminant analysis (PLS-DA) and orthogonal partial least squares discriminant analysis (OPLS-DA) of the metabolomic data were carried out using SIMCA-P 14.0 software (Umetrics, Umeå, Sweden). Heatmaps and upset plot were generated using TBtools(version 2.309). Volcano plot, hierarchical cluster analysis (HCA), Nightingale diagram, stacked bar chart, and mantel test were created using R Studio (version 4.4.1).

## 3. Results and Discussion

### 3.1. Inoculation with AN at 25–37 °C Contributes to Form the Sensory Quality of Dark Tea

Fermentation temperature is a key factor affecting the flavor quality of dark tea, as it influences enzyme activity and the succession of microbial communities [5]. The sensory qualities of teas fermented by AN under different temperatures are shown in Table 1. The sensory quality of dark tea first increased and then decreased with rising temperature, with AN-inoculated teas at low temperatures (25–37 °C) exhibiting superior attributes.

Previous studies indicate that microbial richness and diversity decline with rising fermentation temperatures [5]. AN is the dominant microorganism in the pile fermentation of dark tea. In the early stages of pile fermentation of Pu-erh tea, the number of AN increased rapidly and gradually decreased in the middle and later stages [16]. These results imply that AN growth is likely inhibited at temperatures exceeding 40 °C. Because AN growth is inhibited at high temperatures, samples like the control (CK) were primarily influenced by moist heat reactions, which significantly reduced the rate of quality component transformation and hindered the formation of characteristic quality in dark tea. This explains the low scores of CK, AN40, and AN45 (Table 1). A recent study [17] demonstrated that, compared to traditional fermentation, inoculating *Debaryomyces hansenii* PETY002 improved the quality of Pu-erh tea, resulting in a deeper infusion color, a richer taste, and a distinct fungal aroma, which is basically consistent with the results of this study (Table 1). In summary, fermenting dark tea with AN at 25–37 °C is more conducive to enhancing the flavor quality of the tea.

### 3.2. Dynamic Change in Major Quality Compounds at Different Fermentation Temperatures

Catechins are the main components of tea polyphenols (TPs) and contribute to the bitter and astringent taste of tea infusion. In this study, the contents of TPs and most catechin monomers in CK and AN45 were significantly higher than those in other tea samples (*p* < 0.05; Table 2). At 45 °C, the growth of microorganisms such as AN may be inhibited [18]. Consequently, the metabolic activities in CK and AN45 are primarily driven by moist-heat effects, resulting in slower metabolic efficiency. Microorganisms exhibit a faster growth rate at 25–40 °C, thereby accelerating the utilization of carbon sources such as polyphenols [19]. Additionally, this temperature range may be more conducive to activating polyphenol oxidase (PPO) and peroxidase (POD) [20], thus promoting the oxidative polymerization of TPs [14]. This explains the high levels of polyphenols and the stronger astringency in CK and AN45, which aligns with the sensory evaluation (Table 1). Theaflavins (TFs), thearubigins (TRs), and theabrownins (TBs) are typical contributors to the color and taste of tea infusion. In this study, the contents of TFs and TRs in CK and AN45 were also higher than those in other tea samples, while TBs showed the opposite trend (Table 2). TFs determine the brightness of tea infusion, whereas TRs influence its redness. TBs are produced through the further oxidative polymerization of TFs and TRs [21]. These results further confirm that inoculating AN at an appropriate fermentation temperature (30–40 °C) promotes the oxidative polymerization of polyphenols. Amino acids not only shape the taste of tea infusion but also serve as important precursors for aroma compounds [14]. In this study, the free amino acid (FAA) content in AN37 and AN40 was significantly higher than that in other tea samples (*p* < 0.05; Table 2). This is likely because the temperature facilitated protease-mediated protein hydrolysis, thus causing amino acid production to outpace its consumption by microbial growth. Soluble sugars (SSs) are responsible for the sweetness of tea infusion. In this study, the SS content in CK and AN45 was significantly higher than that in other tea samples (*p* < 0.05; Table 2), which may also be attributed to the substantial consumption of SSs as carbon sources by microorganisms at 25–40 °C. In summary, inoculating AN at 30–40 °C enhances microbial utilization of carbon sources such as TPs and SSs in tea leaves, while microbial metabolic activity intensifies alongside its growth. Furthermore, this temperature range not only stimulates microbial growth but also likely enhances the activity of enzymes involved in substrate conversion, thereby further promoting the oxidative polymerization of polyphenols and the hydrolysis of proteins.

### 3.3. Inoculation with AN at Different Fermentation Temperatures Impacts the Composition of Volatile Compounds in Dark Tea

**Figure 1:**
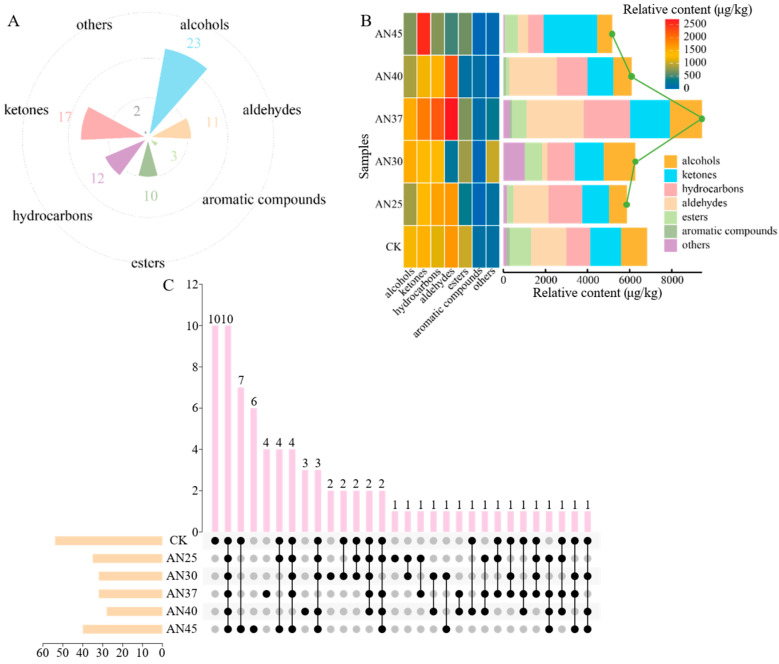
(**A**) The quantity of different types of volatiles in tea leaves under different treatments; (**B**) heatmap and stacked bar chart of various volatile compounds in tea leaves under different treatments; (**C**) common and unique volatile compounds in tea leaves under different treatments.

The aroma quality of tea is determined collectively by diverse volatile compounds. As an indispensable quality factor, the aroma of dark tea directly influences its market value. In this study, 78 volatiles were identified in tea leaves fermented at various temperatures using HS-SPME-GC-MS (Table S1). These volatiles were primarily alcohols, ketones, hydrocarbons, and aldehydes, followed by esters and aromatic compounds (Figure 1A). As the fermentation temperature increased, the total content of volatile compounds in the tea showed a trend of first rising and then falling (Figure 1B). Aldehydes, hydrocarbons, ketones, and alcohols were the dominant categories (Figure 1B), which is consistent with a previous study [22]. Notably, the total volatile content in tea fermented with AN at 37 °C was higher than that at other temperatures (Figure 1B). Generally, 37 °C is the optimal growth temperature for *Aspergillus niger* [6]. This temperature not only favors the metabolic activities of microbial communities such as AN, but may also enhance enzyme activity, thereby promoting glycoside hydrolysis, the degradation of unsaturated fatty acids and amino acids [23], which in turn facilitates the release and accumulation of volatile compounds.

Fermentation temperature influences both the quantity and diversity of volatile compounds in dark tea. In this study, 54 volatiles were detected in CK, 35 in AN25, 32 in both AN30 and AN37, 28 in AN40, and 40 in AN45 (Table S1). This finding indicates that natural fermentation at 30 °C is more conducive to the production of diverse volatiles. Moreover, a total of 10 common volatiles were identified across all tea samples (Figure 1C), including linalool, *D*-limonene, *α*-ionone, methyl salicylate, and 6-methyl-5-hepten-2-one (Table S2). These volatiles are crucial for forming the characteristic aromas of dark tea. For instance, linalool and its oxides are considered important contributors to the fungal aroma of Liupao tea [24]. *D*-limonene, with its lemon-like aroma and low odor threshold, plays a critical role in shaping the distinctive aroma of ripened Pu-erh tea [1]. *α*-Ionone is regarded as a key marker distinguishing raw from ripened Pu-erh tea and a primary component responsible for woody aromas in ripened Pu-erh tea [1]. Methyl salicylate, which has the aroma of holly oil, is recognized as a key aroma-active compound in ripened Pu-erh tea, Liupao tea, Fuzhuan tea, and Qingzhuan tea [1]. In addition, the number of unique volatiles in CK, AN25, AN30, AN37, AN40, and AN45 was 10, 1, 2, 4, 3, and 6, respectively (Figure 1C). In summary, fermentation with inoculated AN reduces the variety of volatile compounds in tea leaves. Different fermentation temperatures affect the composition of volatiles in AN-fermented tea, with 37 °C being the most favorable for the accumulation of volatile content.

### 3.4. The Difference in Volatiles at Different Fermentation Temperatures

**Figure 2:**
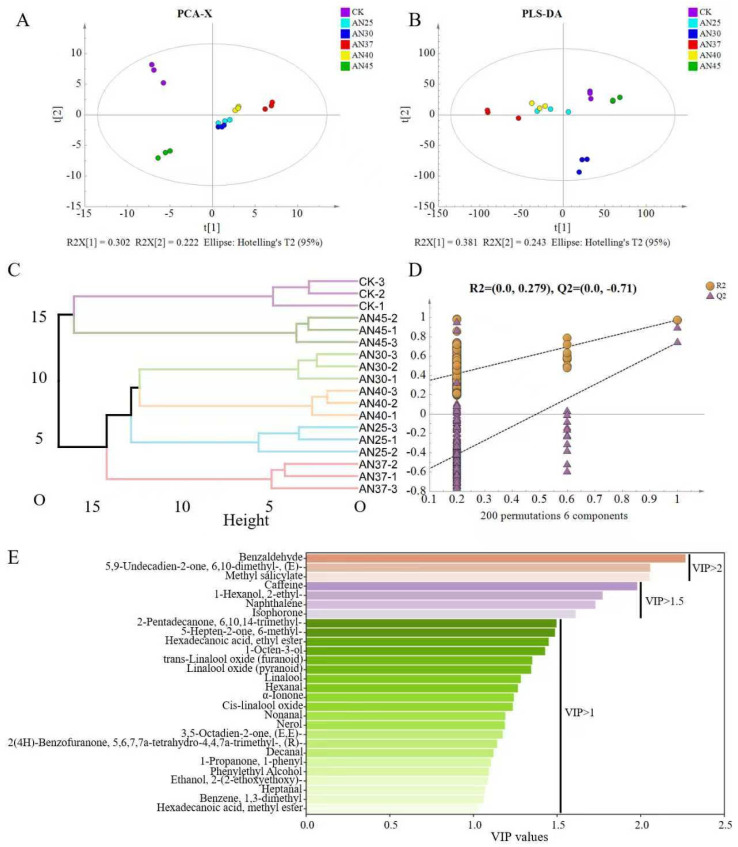
(**A**) Principal component analysis (PCA); (**B**) partial least squares discriminant analysis; (**C**) hierarchical cluster analysis (HCA); (**D**) permutation plot; (**E**) the variable importance in projection (VIP) plot of volatiles.

To explore the differences in volatile metabolic profiles among the treatments, multivariate statistical analysis was conducted. The PCA results showed that the tea samples were separated, indicating differences in the volatile metabolites among these samples (Figure 2A). The PLS-DA results also demonstrated differences in volatile metabolites among the tea samples (Figure 2B), consistent with the PCA results. The HCA results showed that AN25, AN30, and AN40 clustered together, indicating a more similar volatile metabolite composition among these samples (Figure 2C). These results suggest that inoculated fermentation with AN at temperatures of 25–30 °C and 40 °C has a similar impact on the volatiles in tea leaves. The permutation test results of PLS-DA confirmed the validity of the model (Figure 2D). Based on a VIP analysis of PLS-DA, 28 differential volatiles were identified (VIP > 1, Figure 2E), revealing the profound influence of AN and fermentation temperature on volatiles. These differential volatiles include benzaldehyde, methyl salicylate, trans-linalool oxide (furanoid), linalool, hexanal, *α*-ionone, cis-linalool oxide, nerol, and phenylethyl alcohol—many of which are established crucial aroma-active compounds in tea leaves.

These differential volatiles exhibited significant changes under different fermentation temperatures (Figure S1, Table S3). Notably, most key aroma-active compounds accumulated to their highest levels at 37 °C (Figure S1). These compounds, which included benzaldehyde (A1), 6-methyl-5-hepten-2-one, (A9), 6-ethenyltetrahydro-2,2,6-trimethyl-2H-pyran-3-ol (linalool oxide (pyranoid)) (A13), linalool (A14), and α-ionone (A16), may be important components contributing to the fungal aroma of the AN37 sample. The content of nerol (A19) peaked in AN25, while *trans*-linalool oxide (furanoid) (A12) peaked in AN30. Hexanal (A15) and *cis*-linalool oxide (A17) peaked in AN40. Most ketones, such as (*E*)-6,10-dimethyl-5,9-undecadien-2-one (A2), (*E*,*E*)-3,5-octadien-2-one (A20), 2(4H)-benzofuranone, 5,6,7,7a-tetrahydro-4,4,7a-trimethyl-,-(A21), and 1-propanone, 1-phenyl (A23), reached their highest levels in AN45, indicating that higher fermentation temperatures favor the accumulation of these ketones. Additionally, methyl salicylate (A3), decanal (A22), phenylethyl alcohol (A24), and heptanal (A26) peaked in CK. The results suggest that high levels of benzaldehyde, α-Ionone, 6-methyl-5-hepten-2-one, linalool and its oxides are associated with the formation of a high-quality tea aroma.

### 3.5. Key Volatiles in Tea at Different Fermentation Temperatures

The overall aroma contribution of tea is determined by the concentrations of aroma compounds and their odor thresholds. Typically, volatiles with an rOAV > 1 are considered to significantly contribute to the characteristic aromas [15]. To further identify the key aroma-active compounds in teas fermented with AN at different temperatures, 18 volatiles were selected as potential aroma-active compounds (Table S4) based on previously reported odor thresholds [15,25,26]. These aroma-active compounds included benzaldehyde, methyl salicylate, linalool, *trans*-linalool oxide (furanoid), *cis*-linalool oxide, nerol, and α-ionone. Specifically, 13 key aroma-active compounds (rOAV > 1) were found in the CK, with nonanal (rOAV = 163.99), 1-octen-3-ol (rOAV = 158.81), decanal (rOAV = 56.4), and heptanal (rOAV = 53.6) being the main aroma contributors. In AN25, 10 key aroma-active compounds (rOAV > 1) were identified, with nonanal (rOAV = 98.76), *α*-ionone (rOAV = 86.48), and benzaldehyde (rOAV = 54.67) as the main aroma contributors. In AN30, 11 key aroma-active compounds (rOAV > 1) were found, with 1-octen-3-ol (rOAV = 461.7), ethanol, 2-(2-ethoxyethoxy)- (rOAV = 269.73), nonanal (rOAV = 136.25), and *α*-ionone (rOAV = 93.91) as the main aroma contributors. In AN37, 9 key aroma-active compounds (rOAV > 1) were identified, with nonanal (rOAV = 201.34), α-ionone (rOAV = 138.2), linalool (rOAV = 92.58), and benzaldehyde (rOAV = 86.23) as the main contributors. In AN40, 11 key aroma-active compounds (rOAV > 1) were found, with nonanal (rOAV = 265.92), α-ionone (rOAV = 63.28), and benzaldehyde (rOAV = 62.77) as the main contributors. In AN45, 9 key aroma-active compounds (rOAV > 1) were identified, with α-ionone (rOAV = 55.27), linalool (rOAV = 24.75), and benzaldehyde (rOAV = 16.81) as the main contributors. In conclusion, AN inoculation at different temperatures alters the composition and proportion of key volatiles, thereby shaping distinct aromatic quality characteristics. Notably, nonanal, *α*-ionone, and benzaldehyde were identified as the primary aroma-active substances across different fermentation conditions.

### 3.6. Formation Mechanism of Key Volatile Compounds

**Figure 3:**
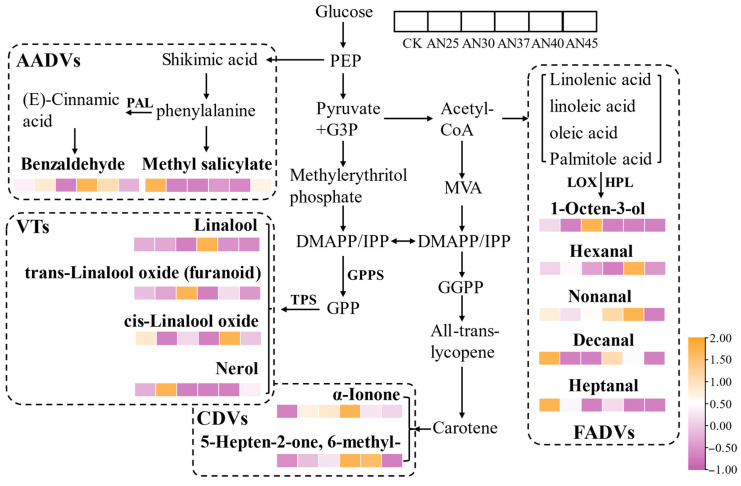
The formation pathway of key volatiles.

Based on different precursors, these key volatiles can be categorized into fatty acid-derived volatiles (FADVs), volatile terpenes (VTs), carotenoid-derived volatiles (CDVs), and amino acid-derived volatiles (AADVs) (Figure 3).

FADVs: Aldehydes are primarily derived from the Strecker degradation and the degradation of unsaturated fatty acids [27]. In these pathways, volatiles are mainly formed through the oxidative degradation of unsaturated fatty acids, such as linoleic and linolenic acids. Under the action of lipoxygenases (LOXs), hydroperoxide lyases (HPLs), and alcohol dehydrogenases (ADHs), these fatty acids generate volatiles including hexanal, nonanal, decanal, heptanal, and 1-octen-3-ol. Due to their low odor thresholds, hexanal, nonanal, heptanal, and 1-octen-3-ol are key contributors to the tea aroma profile [25]. Nonanal, in particular, contributes to the aged, woody, and herbal aromas characteristic of Liupao tea, while decanal is recognized as a key aroma compound in ripe Pu-erh tea due to its high odor intensity [1]. The inoculation with AN for fermentation, as well as different fermentation temperatures, has varying effects on the production of these volatiles. Overall, the contents of these FADVs were lower in AN45 (Figure 3), indicating that higher fermentation temperatures inhibit their accumulation, likely due to the inactivation of enzymes such as LOXs and HPLs [27].

VTs: Linalool and its oxides, and nerol are derived from the monoterpene biosynthesis pathway. These volatiles are typically present in tea as glycoside conjugates and released through the action of glycosidases [1,25]. Linalool oxides contribute to floral and woody aromas [28]. In addition to being released through glycoside hydrolysis, linalool oxides can also be formed from linalool under the high-temperature and high-humidity conditions of fermentation [1]. Overall, linalool and its oxides were present at higher levels at fermentation temperatures between 30 and 40 °C (Figure 3), suggesting that this range may be more favorable for glycosidase activity. However, prolonged exposure to high-temperature and high-humidity environments may lead to the extensive degradation of VTs or their re-conjugation into non-volatile compounds [25], explaining the lower VT content in AN45 (Figure 3).

CDVs and AADVs: *α*-ionone is a degradation product of carotenoids. In addition to its floral aroma, which is similar to that of *β*-ionone, *α*-ionone is particularly notable for its woody scent [1]. Following AN inoculation, the content of *α*-ionone peaked at 37 °C and then decreased with further increases in fermentation temperature. Additionally, the content of *α*-ionone was lowest at 30 °C under natural fermentation conditions (Figure 3). The compound 6-methyl-5-hepten-2-one, known for its orange aroma, is also derived from the carotenoids degradation [1]. Notably, AN inoculation, particularly at 37–40 °C, promoted the accumulation of this compound (Figure 3). These results suggest that AN inoculation promotes carotenoid degradation, with 37 °C being optimal for the accumulation of carotenoid derivatives. Methyl salicylate, a key aroma component contributing to the betel nut and fungal aromas of Liupao tea [29], can be synthesized through the catalysis of salicylic acid carboxyl methyltransferase or formed by the hydrolysis of glycosides under the action of microbial glycosidases [30]. Dominant microorganisms in the long-term storage and post-fermentation process can lead to the formation of methyl salicylate in dark tea [4]. Notably, methyl salicylate content was generally lower in AN-fermented teas and highest in the CK (Figure 3), indicating that AN fermentation inhibits its formation. Benzaldehyde is recognized as a key aroma-active compound contributing to the fungal aroma in Liupao tea [29]. Prunasin is rapidly hydrolyzed to form benzaldehyde under the action of *β*-glucosidase (prunasin hydrolase) and mandelonitrile lyase [31]. During the post-fermentation of tea leaves, certain microorganisms may produce *β*-glucosidase (prunasin hydrolase) and mandelonitrile lyase, thus promoting the formation of benzaldehyde [1]. In contrast to methyl salicylate, the content of benzaldehyde is generally higher in AN-fermented teas, especially at 37 °C (Figure 3). In summary, lipids and glycosides serve as key precursors for aroma formation in AN-inoculated dark tea at various fermentation temperatures. The fermentation temperature, particularly around 37 °C, critically regulates this process by influencing microbial activity, thereby shaping the final aromatic profile.

### 3.7. Inoculation with AN at Different Fermentation Temperatures Impacts the Composition of Non-Volatile Compounds in Dark Tea

**Figure 4:**
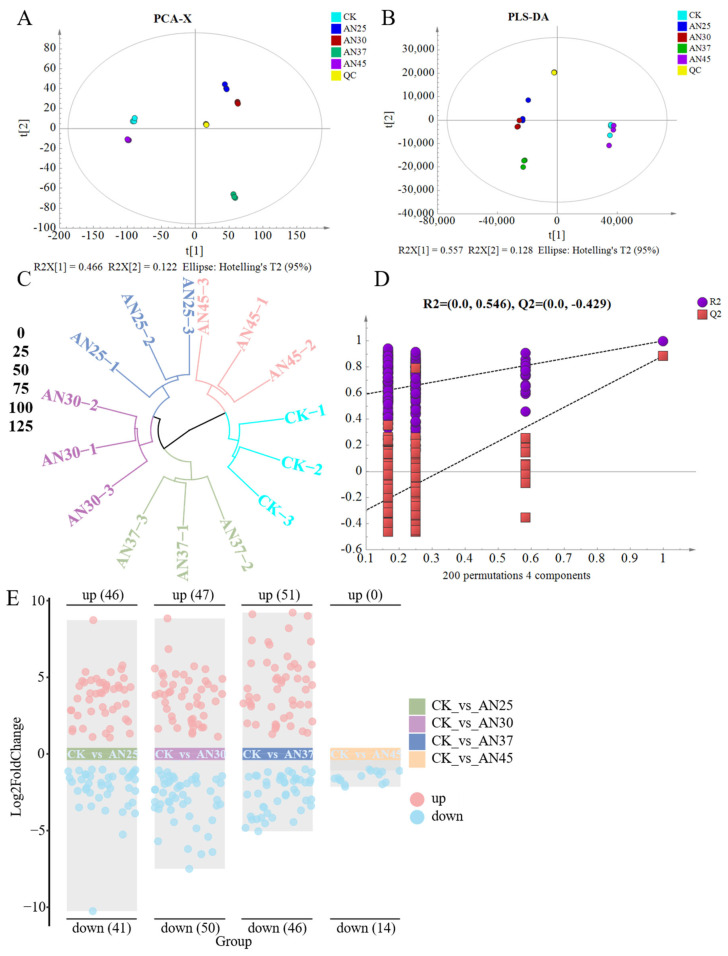
(**A**) PCA; (**B**) PLS-DA; (**C**) HCA; (**D**) permutation plot; (**E**) volcano plot of metabolites in each comparison group.

Taste is the most crucial factor in evaluating the quality of tea, which is collectively determined by various non-volatile metabolites. To investigate the changes in these metabolites in tea under different fermentation temperatures, a combination of LC-MS-based metabolomics and multivariate analysis was employed. A total of 4941 metabolites were identified across CK, AN25, AN30, AN37, and AN45 in both positive and negative ion modes (see Table S5 for details). Multivariate statistical analyses were performed on these metabolites to gain a comprehensive understanding of the variations. The PCA clearly distinguished all sample groups (Figure 4A), indicating that AN inoculation and fermentation temperatures alter the overall metabolite profiles. The QC samples clustered tightly near the origin, demonstrating excellent system stability and data reliability. The PLS-DA results also revealed distinct metabolite profiles among the samples (Figure 4B), consistent with the PCA results. The HCA results grouped these samples into three clusters: CK and AN45 as one group, AN37 as another, and AN25 and AN30 as the third (Figure 4C). This clustering suggests that the non-volatile metabolic profiles of CK and AN45, as well as AN25 and AN30, are more similar to each other. The permutation test results of PLS-DA confirmed the validity of the model (Figure 4D).

Using the OPLS-DA model (Figure S2), pairwise comparisons of metabolites between CK and AN25, CK and AN30, CK and AN37, and CK and AN45 were conducted to elucidate the differential metabolic changes in tea under AN fermentation at different fermentation temperatures. A total of 146 non-volatile differential metabolites were identified based on the criteria of adjusted *p* < 0.01, VIP > 2, fold change > 2, and fold change < 0.5. These differential metabolites included organic acids and their derivatives (26.71%), amino acids and their metabolites (23.97%), flavonoids (21.92%), alcohols and amines (7.53%), nucleotides and their metabolites (6.85%), carbohydrates and their metabolites (3.42%), alkaloids (0.68%), lignans and coumarins (0.68%), and phenolic acids (0.68%) (Figure S3). In the comparison between CK and AN25, 87 non-volatile differential metabolites were identified, including 46 upregulated and 41 downregulated metabolites (Figure 4E, Table S6). In the comparison between CK and AN30, 97 non-volatile differential metabolites were identified, including 47 upregulated and 50 downregulated metabolites (Figure 4E, Table S7). In the comparison between CK and AN37, 97 non-volatile differential metabolites were identified, including 51 upregulated and 46 downregulated metabolites (Figure 4E, Table S8). Finally, in the comparison between CK and AN45, 14 non-volatile differential metabolites were identified, all of which were downregulated (Figure 4E, Table S9). KEGG pathway enrichment analysis was performed on these differential metabolites (Figure S4). The results indicated that these differential metabolites were primarily enriched in amino acid metabolic pathways, such as glycine, serine, and threonine metabolism, lysine biosynthesis, phenylalanine metabolism, phenylalanine, tyrosine, and tryptophan biosynthesis, and cysteine and methionine metabolism. They were also enriched in carbohydrate metabolic pathways, such as galactose metabolism and starch and sucrose metabolism. Additionally, flavone and flavonol biosynthesis and flavonoid biosynthesis were also enriched. In summary, AN-inoculated fermentation at different temperatures influences the composition of non-volatile metabolites in tea, primarily affecting organic acids and their derivatives, amino acids and their metabolites, and flavonoids.

#### 3.7.1. Flavonoids

**Figure 5:**
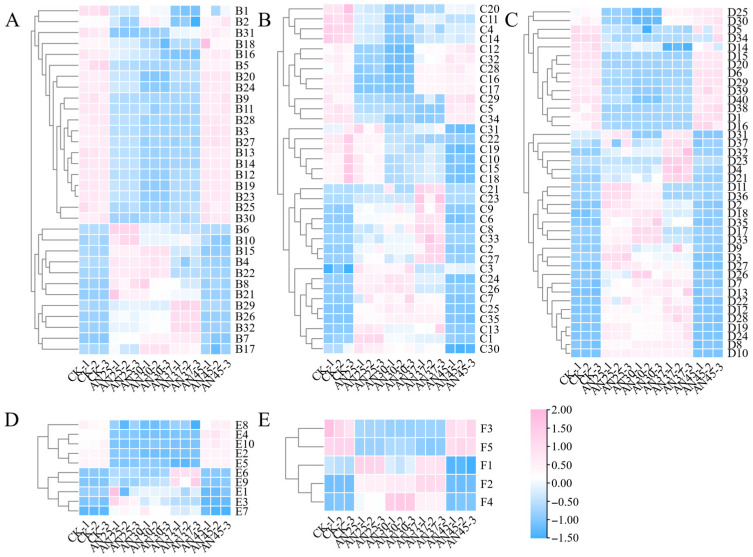
(**A**) Heatmap of flavonoid metabolites; (**B**) heatmap of amino acid and its metabolites; (**C**) heatmap of phenolic acids, organic acid and its derivatives; (**D**) heatmap of nucleotide and its metabolites; (**E**) heatmap of carbohydrates and its metabolites.

Flavonoids play a crucial role in shaping the color and flavor quality of tea products [26]. In this study, 32 flavonoids were identified as differential metabolites, including catechins and their derivatives, proanthocyanidins and their derivatives, quercetin glycosides, and kaempferol glycosides (Table S10). Overall, the majority of flavonoids were present in higher levels in CK and AN45. AN37 exhibited the lowest abundance of flavonoids, with only quercetin 3-*O*-rhamnoside-7-*O*-glucoside (B29), quercetin 3-(2Gal-apiosylrobinobioside) (B26), and xanthotoxol arabinoside (B32) showing the highest abundance (Figure 5A). Catechins, the main components of tea polyphenols, are the primary bitter and astringent compounds in tea [26]. In this study, catechins and their derivatives, such as (-)-gallocatechin (GC; B1), epigallocatechin gallate (EGCG; B14), epicatechin (4b→8) epigallocatechin 3-*O*-gallate (B12), epigallocatechin 3-*O*-(3-*O*-methyl) gallate (B13), and gallocatechin-(4*α*→8)-epigallocatechin (B16) were significantly higher in CK and AN45 (*p* < 0.05) (Figure 5A). The above-mentioned compounds primarily contribute bitter and astringent tastes, and their reduction helps improve the flavor of the tea infusion [32], which may be an important reason for the superior taste quality of AN37. (Table 1). AN exhibits its highest growth rate at 37 °C [18], leading to substantial consumption of carbon sources by the microorganisms. Furthermore, this temperature is likely more favorable for enhancing enzymatic activity [23], thereby accelerating the enzymatic oxidation, polymerization, and hydrolysis of flavonoids. The hydrolysis products, such as catechins and gallic acid, are subsequently further utilized by the microorganisms [33]. This is the result of the combined effects of “optimized growth conditions” and “activated specific pathways.”

#### 3.7.2. Amino Acid and Its Metabolites

Amino acids are key taste compounds and aroma precursors in tea and also exert physiological functions [34]. In this study, 35 amino acids and their metabolites, predominantly polymers, were identified as differential metabolites (Table S11). Overall, CK and AN37 showed higher levels of amino acids and their metabolites (Figure 5B), suggesting that natural fermentation at 30 °C and AN fermentation at 37 °C are more favorable for protein hydrolysis. The 37 °C condition likely promotes protein hydrolysis, and the resulting hydrolytic products serve as a nitrogen source to support the growth and reproduction of microorganisms such as AN. Meanwhile, AN may secrete proteases to further enhance protein hydrolysis [35]. As a result, AN exhibits a higher growth rate at 37 °C, accompanied by abundant amino acids and their metabolites.

#### 3.7.3. Phenolic Acids and Organic Acid and Its Derivatives

Phenolic acids and organic acids are critical components in tea, influencing its sensory characteristics [34]. In this study, organic acids and their derivatives constituted the largest category among differential metabolites, with 39 compounds identified (Table S12, including one phenolic acid). This indicates that AN fermentation at different temperatures primarily affects the levels of organic acids and their derivatives. Most of these compounds were more abundant in AN-inoculated fermentation at 25–37 °C (Figure 5C). Notably, some common phenolic acids or organic acids, such as 4-glucogallic acid (D16), quinic acid (D39), and 3-*p*-coumaroylquinic acid (D40), showed significantly higher abundance in CK and AN45 (*p* < 0.05; Figure 5C). Studies suggest that caffeoylquinic acids may degrade into quinic acid during fermentation [36], and quinic acid potentially serves as a carbon source for microbial proliferation [19]. Furthermore, caffeoylquinic acids can condense with catechins through POD catalysis to form theaflavin-like compounds [36]. These findings indicate that temperatures between 25 and 37 °C accelerate the utilization of compounds such as quinic acid, 3-*p*-coumaroylquinic acid, and 4-glucogallic acid by microorganisms like AN to support their growth. Simultaneously, this temperature range also promoted the oxidative polymerization of these compounds. Together, these processes contributed to the reduction in their content.

#### 3.7.4. Nucleotide and Its Metabolites

Nucleotides and their derivatives not only possess significant functional and nutritional properties but also impart umami and a mellow, thick taste to tea [37]. In this study, ten nucleotides and their metabolites were identified as differential metabolites (Table S13). Although nucleotides are present at lower concentrations than alkaloids and amino acids, they enhance tea flavor by interacting with taste receptors [38]. For instance, inosine 5′-monophosphate (IMP) and guanosine 5′-monophosphate (GMP) contribute to umami, while adenosine 5′-monophosphate (AMP) enhances the sweetness of tea infusion [39]. Overall, the abundance of most nucleotides and their metabolites was significantly higher in CK and AN45 compared to other samples (*p* < 0.05), such as 6-methylthioinosine (E2), adenosine (E4), cordycepin (E5), photrexa (E8), and toyocamycin (E10) (Figure 5D). These results suggest that fermentation with AN at 25–37 °C may also accelerate the utilization of nucleotides and their metabolites by microorganisms such as AN to support their growth and reproduction. Additionally, some decomposition products of nucleotides and their metabolites may contribute to enhancing the flavor quality of tea. For instance, purine nucleotides such as IMP, GMP, and AMP can be degraded into hypoxanthine [38], which, along with other purine alkaloids, may mask off-flavors in tea [40].

#### 3.7.5. Carbohydrates and Its Metabolites

Carbohydrates help enhance the viscosity of tea infusion and are a primary source of its sweet and mellow taste [26]. Additionally, the Maillard reaction between carbohydrates and amino acids contributes to the aroma quality of tea [41]. In this study, five carbohydrates and their metabolites were identified, including melezitose (F3), melibiose (F4), and sucrose (F5) (Table S14). These carbohydrates may serve as carbon sources for the growth and reproduction of microorganisms. Furthermore, hydrolysis products of polysaccharides, such as glucose, can enhance the sweetness of tea infusion and alleviate bitterness and astringency. In this study, the levels of melezitose (F3) and sucrose (F5) were significantly higher in CK and AN45 than in other samples (*p* < 0.05; Figure 5E). These results suggest that temperatures between 25 and 37 °C promote the utilization of carbon sources such as melezitose and sucrose by microorganisms such as AN to support their growth and reproduction.

In summary, the accumulation patterns of metabolites in tea leaves result from the combined effects of “optimized growth conditions” and “activated specific biochemical pathways.” At 37 °C, AN exhibits a higher growth rate, thereby accelerating its utilization of carbon and nitrogen sources such as tea polyphenols, amino acids, nucleotides, and sugars, which in turn enhances microbial metabolic activity. Furthermore, this temperature may favor increased enzyme activity, thereby further promoting the progression of reactions within relevant metabolic pathways, such as oxidation, polymerization, and hydrolysis. In contrast, the high temperature of 45 °C may inhibit the growth of microorganisms such as AN, resulting in metabolite levels that are relatively similar to those in CK.

### 3.8. Correlation Between Key Volatile and Non-Volatile Compounds and Flavor Factors

**Figure 6:**
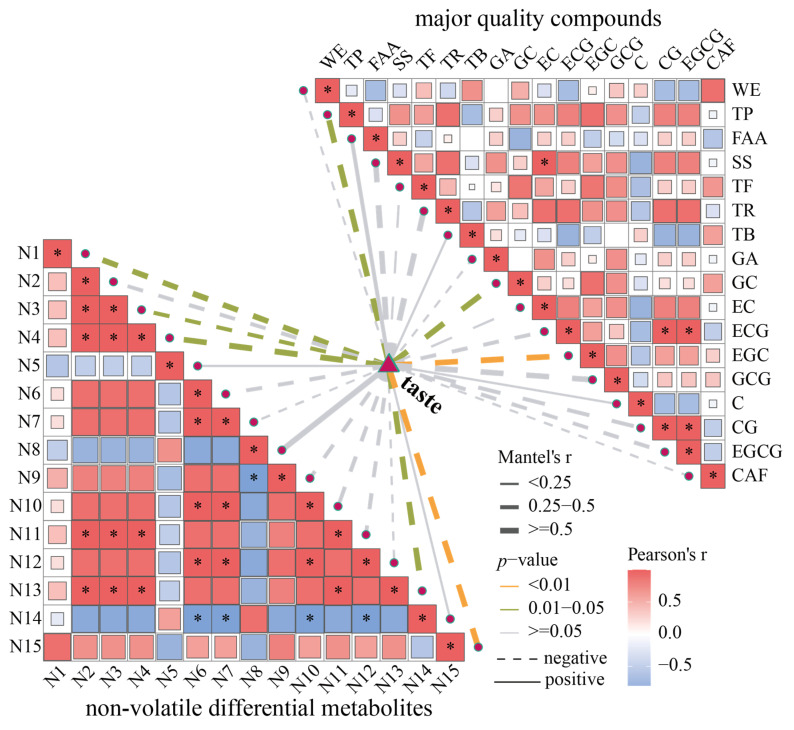
The correlation heat map of the main quality compounds, non-volatile differential metabolites and taste scores. * indicates significant correlation (*p* < 0.05).

To explore potential volatile compounds contributing to aroma quality of tea, a correlation analysis was performed between the 18 key volatiles and aroma scores. As shown in Figure S5, most volatile compounds exhibited no significant correlation with aroma scores. Notably, cis-linalool oxide (V11) and benzene, 1,3-dimethyl (V18) were significantly negatively correlated with the aroma score (*p* < 0.05). Additionally, 1-octen-3-ol (V6), trans-linalool oxide (furanoid) (V7), linalool (V8), *α*-ionone (V10), 2,6-octadien-1-ol, 3,7-dimethyl-, (*Z*)- (V13), 3,5-octadien-2-one, (*E*,*E*)- (V14), and ethanol, 2-(2-ethoxyethoxy)- (V16) showed positive correlations with aroma scores. These compounds primarily contribute floral, fruity, creamy, and other pleasant aromas (Table S4), which may help form the complex fungal aroma [42], thereby enhancing the overall aroma quality of the tea. Isophorone (V4), 5-hepten-2-one, 6-methyl- (V5), hexanal (V9), nonanal (V12), and decanal (V15) showed negative correlations with aroma scores. These volatiles contribute green-like odors (Table S4), indicating that this type of aroma is detrimental to the formation of the fungal aroma in tea. Similarly, a correlation analysis of some typical non-volatile differential metabolites and main quality compounds together with the taste score was conducted. As shown in Figure 6, most of the non-volatile differential metabolites exhibited strong positive correlations with each other, suggesting a synergistic effect on taste. Among the differential metabolites, (-)-GC (N1), epigallocatechin 3-*O*-(3-*O*-methyl) gallate (N3), 3′-galloylprodelphinidin B2 (N4), and melezitose (N13) showed significant negative correlations with taste scores (*p* < 0.05). Similarly, TP and GC among the main quality compounds were also significantly negatively correlated with the taste score (*p* < 0.05). Notably, sucrose (N15) and epigallocatechin (EGC) exhibited an extremely significant negative correlation with taste scores (*p* < 0.01). TPs, primarily catechins, are the most abundant quality compounds in tea infusion and contribute to bitter and astringent taste. According to Jin et al. [21], GC and EGC show a significant negative correlation with the umami, sweetness and mellowness of tea infusion (*p* < 0.05). These results indicate that GC and EGC can be used as marker compounds for evaluating taste quality. A recent study by [43] found that epicatechin 3-(3-*O*-methylgallate) has a strong positive correlation with the bitterness of tea infusion, while proanthocyanidins and their derivatives exhibit strong positive correlations with bitterness and astringency but strong negative correlations with sweet and mellow taste. Although sugars generally enhance sweetness, one study found that sucrose was significantly positively correlated with bitterness and astringency [44]. Polysaccharides may be hydrolyzed during processing, and their hydrolysis products (such as glucose) could contribute to the mouthfeel of tea infusion. In conclusion, fermentation temperature influences the composition and proportion of compounds, thereby impacting tea flavor. The taste quality of tea was closely related to TPs such as GC, EGC, and epigallocatechin 3-*O*-(3-*O*-methyl) gallate, as well as polysaccharides such as melezitose and sucrose, and shows a negative correlation. However, aroma and taste quality of tea are determined by the mutual coordination and interaction of multiple flavor compounds, rather than a single compound. Future studies could further elucidate the roles of key flavor substances through aroma and taste reconstitution and omission experiments.

### 3.9. Analysis of Industrial Scalability and Sustainability

To achieve the industrial-scale application of the 37 °C inoculated fermentation process, several engineering and sustainability challenges must be addressed. Industrial implementation of precise and constant 37 °C fermentation requires integrated engineering approaches. Modular fermentation tanks or temperature-controlled fermentation chambers with built-in regulated hot-air systems can be employed for dynamic temperature control. A pile height of 1.0–1.5 m, combined with automated aeration, ensures uniform fermentation and avoids localized overheating issues common in traditional pile fermentation. Compared to traditional processes relying on microbial self-heating, the proposed method requires additional energy input for temperature control. However, the fermentation cycle can be significantly shortened from several tens of days to just a few days, increasing production capacity and improving fermentation success rates, which may offer greater overall energy efficiency advantages. Furthermore, the use of standardized AN starter cultures ensures consistent microbial initiation, significantly reducing batch-to-batch quality variability, minimizing raw material waste and energy loss due to fermentation failures or inconsistent quality, and enhancing production stability. The core environmental impact of the process lies in the energy consumption for temperature control. Future optimization could involve technologies such as waste heat recovery to improve energy efficiency, providing a feasible pathway toward green and intensive production in the dark tea industry.

## 4. Conclusions

This study revealed the quality characteristics of dark tea fermented with AN at different temperatures and their effects on volatile and non-volatile compounds. Sensory evaluation indicated that 25–37 °C fermentation with AN was conducive to tea quality formation. HS-SPME-GC-MS analysis identified 78 volatiles, with the highest total content and the highest levels of most key aroma compounds found in the tea fermented with AN at 37 °C. Eighteen key volatiles were screened based on rOAV > 1 and VIP > 1, with nonanal, *α*-ionone, and benzaldehyde identified as the main aroma-active compounds. The composition and proportion of these key volatiles were primarily regulated through pathways involving the degradation of unsaturated fatty acids and monoterpene synthesis. A total of 146 non-volatile differential metabolites were detected by LC-MS, primarily comprising organic acids and their derivatives, amino acids and their metabolites, and flavonoids. At 25–37 °C, the growth rate of AN is relatively high, leading to accelerated utilization of carbon and nitrogen sources in tea leaves such as tea polyphenols, amino acids, and nucleotides. Microbial metabolic activity also intensifies alongside its growth. Moreover, this temperature range likely optimizes the activity and stability of enzymes directly involved in the conversion of tea substrates and flavor formation, thereby promoting hydrolysis, oxidation, polymerization, and degradation reactions of substances such as proteins, tea polyphenols, and nucleotides. These changes improved the taste of the tea infusion. This research provides actionable insights for enhancing the sensory quality, production efficiency, and consistency of dark tea. By adopting temperature-controlled fermentation at 37 °C with AN and leveraging the identified biochemical pathways, the dark tea industry can advance toward more standardized, high-quality, and consumer-oriented products.

## Figures and Tables

**Table 1:** The sensory qualities of dark tea fermented by *Aspergillus niger* under different temperatures.

Sample	Infusion Color (Score)	Aroma (Score)	Taste (Score)	Residue Leaves (Score)	Total Score
CK	green, yellow (84.00 ± 1.00 ab)	mellow (74.67 ± 1.53 c)	astringency (75.33 ± 0.58 d)	green, brown, and hard (70.00 ± 0.00 d)	75.87 ± 0.88 d
AN25	dark orange (81.33 ± 0.58 cd)	mold, mushroom (84.67 ± 1.15 a)	fungal taste (84.00 ± 0.00 b)	bluish brown, soft (84.00 ± 1.00 b)	83.83 ± 0.58 a
AN30	orange, brown (80.00 ± 0.00 d)	heavy, fungal smell (83.33 ± 0.58 a)	fungal taste, mellow (81.33 ± 1.15 c)	bluish brown, soft (82.00 ± 0.00 c)	81.9 ± 0.59 b
AN37	orange (83.00 ± 1.00 bc)	fungal smell (80.00 ± 0.00 b)	fungal taste (88.33 ± 0.58 a)	liver dark reddish, softer (87.33 ± 0.58 a)	84.52 ± 0.43 a
AN40	orange red (86.00 ± 0.00 a)	low, fungal smell (65.00 ± 1.00 d)	fungal taste (70.67 ± 1.15 e)	liver dark (85.33 ± 0.58 b)	72.45 ± 0.84 e
AN45	orange red (74.00 ± 1.00 e)	mellow (80.00 ± 0.00 b)	mellow astringency (80.00 ± 0.00 c)	soft, light brown, red (66.67 ± 1.15 e)	77.77 ± 0.26 c

Note: Different letters in the same column of sensory scores indicate significant differences.

**Table 2:** The content of major quality compounds.

Components	CK	AN25	AN30	AN37	AN40	AN45
WE (mg/g)	439.76 ± 4.90 a	411.10 ± 3.99 b	444.18 ± 1.48 a	413.63 ± 9.55 b	415.40 ± 4.44 b	410.54 ± 2.35 b
TP (mg/g)	136.01 ± 0.56 a	113.24 ± 0.32 c	103.49 ± 0.33 d	100.80 ± 1.13 e	92.96 ± 0.89 f	128.73 ± 0.41 b
FAA (mg/g)	7.35 ± 0.04 d	7.56 ± 0.24 cd	7.13 ± 0.02 e	9.59 ± 0.04 b	10.64 ± 0.04 a	7.68 ± 0.07 c
SS (mg/g)	82.11 ± 0.75 a	58.15 ± 0.58 d	50.91 ± 0.82 e	58.41 ± 0.60 d	64.19 ± 1.16 c	67.35 ± 0.20 b
TF (mg/g)	0.59 ± 0.05 a	0.36 ± 0.06 b	0.54 ± 0.04 a	0.53 ± 0.02 a	0.37 ± 0.04 b	0.54 ± 0.08 a
TR (mg/g)	43.92 ± 0.33 a	20.94 ± 1.33 c	8.95 ± 0.55 f	13.47 ± 0.58 d	11.55 ± 0.40 e	34.91 ± 0.49 b
TB (mg/g)	43.09 ± 0.84 d	42.41 ± 1.01 de	48.69 ± 0.34 b	50.44 ± 0.65 a	46.66 ± 0.28 c	40.81 ± 0.29 e
GA (mg/g)	2.89 ± 0.03 a	2.75 ± 0.06 a	1.33 ± 0.10 c	2.79 ± 0.22 a	2.20 ± 0.12 b	1.89 ± 0.06 b
GC (mg/g)	8.18 ± 0.08 a	6.54 ± 0.15 b	8.11 ± 0.58 a	5.41 ± 0.49 c	5.07 ± 0.33 c	7.63 ± 0.27 a
EC (mg/g)	5.77 ± 0.01 a	4.08 ± 0.10 b	2.37 ± 0.20 c	4.32 ± 0.35 b	1.79 ± 0.13 d	5.72 ± 0.22 a
ECG (mg/g)	9.37 ± 0.07 a	1.62 ± 0.03 b	0.84 ± 0.06 c	1.51 ± 0.12 b	0.59 ± 0.02 c	9.71 ± 0.38 a
EGC (mg/g)	18.74 ± 0.45 a	15.10 ± 0.34 b	15.90 ± 0.78 b	12.41 ± 1.13 c	10.34 ± 0.66 d	16.01 ± 0.73 b
GCG (mg/g)	5.86 ± 0.10 a	0.00 ± 0.00 b	0.00 ± 0.00 b	0.00 ± 0.00 b	0.00 ± 0.00 b	0.00 ± 0.00 b
C (mg/g)	3.05 ± 0.05 cd	7.51 ± 0.17 a	6.16 ± 0.49 b	3.23 ± 0.22 c	3.17 ± 0.22 c	2.44 ± 0.07 d
CG (mg/g)	2.77 ± 0.05 a	2.08 ± 0.05 b	0.48 ± 0.03 d	1.35 ± 0.10 c	0.48 ± 0.02 d	2.88 ± 0.08 a
EGCG (mg/g)	28.55 ± 0.27 a	2.93 ± 0.07 b	1.93 ± 0.16 c	2.17 ± 0.08 bc	1.87 ± 0.13 c	29.23 ± 0.65 a
CAF (mg/g)	13.24 ± 0.16 b	9.04 ± 0.10 c	18.64 ± 0.95 a	12.98 ± 0.46 b	19.69 ± 0.91 a	12.72 ± 0.28 b

Note: The different letters on the same row represent significant differences (*p* < 0.05). Water extract (WE), tea polyphenol (TP), free amino acid (FAA), soluble sugars (SS), theaflavin (TF), thearubigin (TR), theabrownin (TB), gallic acid (GA), gallocatechin (GC), epicatechin (EC), epicatechin gallate (ECG), epigallocatechin (EGC), gallocatechin gallate (GCG), catechin (C), catechin gallate (CG), epigallocatechin gallate (EGCG), caffeine (CAF).

## Data Availability

The original contributions presented in this study are included in the article/Supplementary Materials. Further inquiries can be directed to the corresponding author.

## Supplementary Materials

The following supporting information can be downloaded at https://www.mdpi.com/article/10.3390/foods15030441/s1, Figure S1. The heatmap of differential volatiles in tea leaves under different treatments. The corresponding volatile compounds for the codes are listed in Table S3, with the volatile content expressed as the mean of three replicates. CK refers to tea leaves naturally fermented at 30 °C, while AN25, AN30, AN37, and AN45 represent tea leaves fermented with *Aspergillus niger* (AN) inoculation at 25 °C, 30 °C, 37 °C, and 45 °C, respectively. Figure S2. (A) Orthogonal partial least squares discriminant analysis (OPLS-DA) of non-volatile metabolites between CK and AN25; (B) OPLS-DA of non-volatile metabolites between CK and AN30; (C) OPLS-DA of non-volatile metabolites between CK and AN37; (D) OPLS-DA of non-volatile metabolites between CK and AN45. Figure S3. Proportions of various non-volatile differential metabolites in tea leaves under different treatments. Figure S4. KEGG pathway enrichment analysis of non-volatile differential metabolites in tea leaves under different treatments. Figure S5. Correlation analysis between tea aroma scores and key volatile compound contents under different treatments. The corresponding volatile compounds for the codes (V1, V2, V3) are listed in Table S4. Supplementary Tables S1–S14.
