# Supplementary material for: Influences of Fermentation Temperature on Volatile and Non-Volatile Compound Formation in Dark Tea: Mechanistic Insights Using Aspergillus niger as a Model Organism
Source: Foods. 2026 Jan 26;15(3):441. doi: 10.3390/foods15030441 (PMC12896519; doi:10.3390/foods15030441)
Supplement: Supplementary file 1 [file foods-15-00441-s001.zip › supplementary figures.pdf]

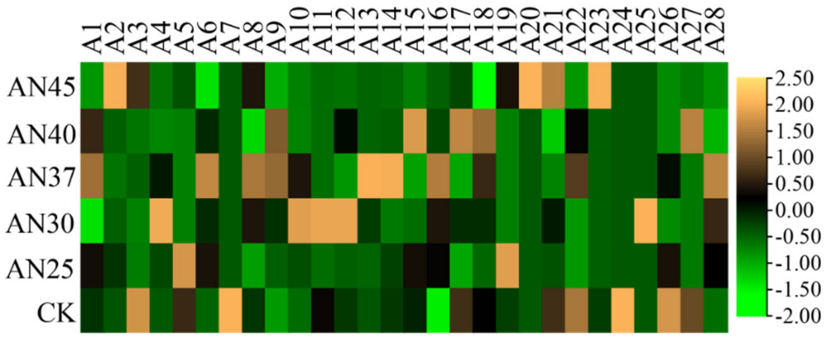

Figure S1. The heatmap of differential volatiles in tea leaves under different treatments. The corresponding volatile compounds for the codes are listed in Table S3, with the volatile content expressed as the mean of three replicates.

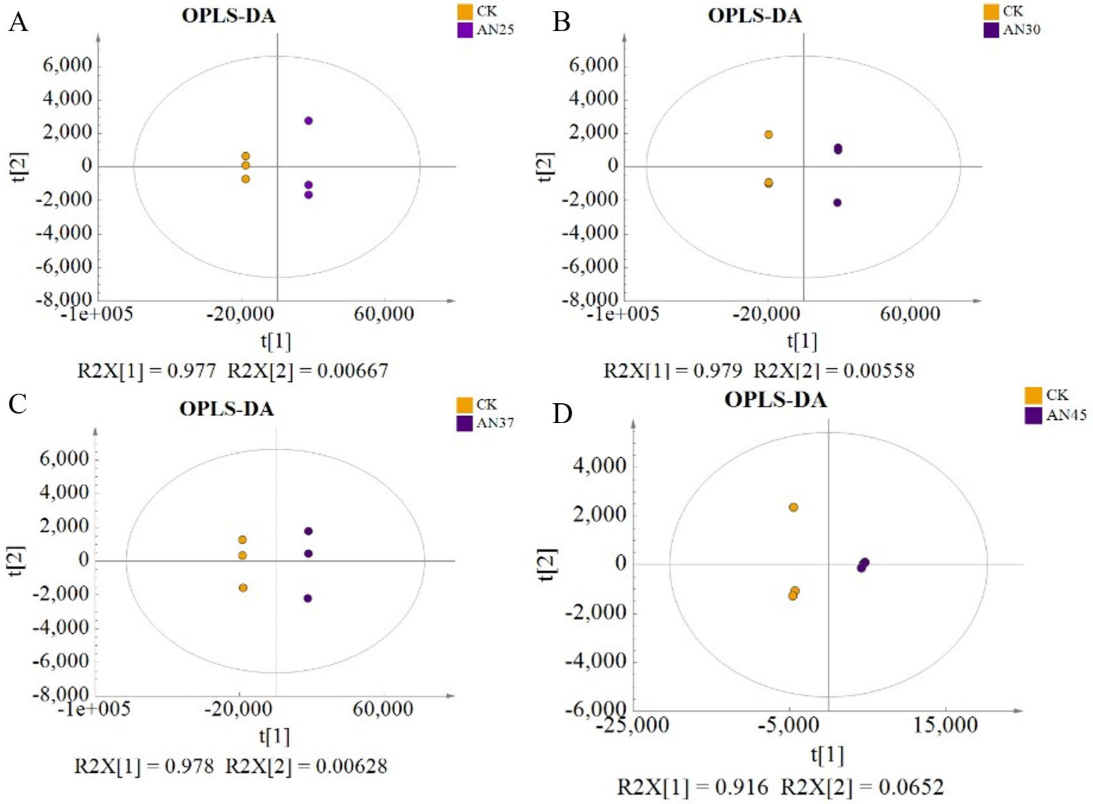

Figure S2. (A) Orthogonal partial least squares discriminant analysis (OPLS-DA) of non-volatile metabolites between CK and AN25; (B) OPLS-DA of non-volatile metabolites between CK and AN30; (C) OPLS-DA of non-volatile metabolites between CK and AN37; (D) OPLS-DA of non-volatile metabolites between CK and AN45. CK refers to tea leaves naturally fermented at 30 °C, while AN25, AN30, AN37, and AN45 represent tea leaves fermented with *Aspergillus niger* (AN) inoculation at 25 °C, 30 °C, 37 °C, and 45 °C, respectively.

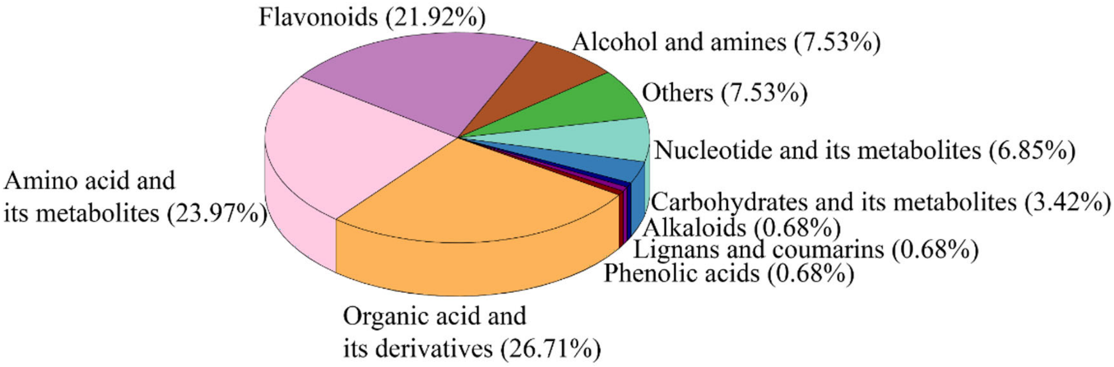

Figure S3. Proportions of various non-volatile differential metabolites in tea leaves under different treatments.

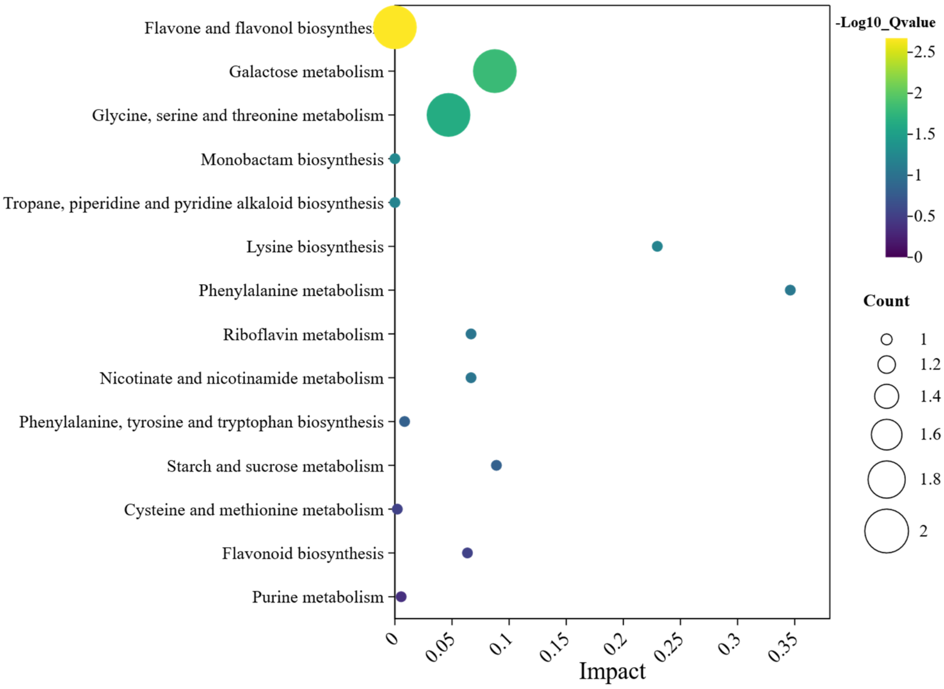

Figure S4. KEGG pathway enrichment analysis of non-volatile differential metabolites in tea leaves under different treatments..

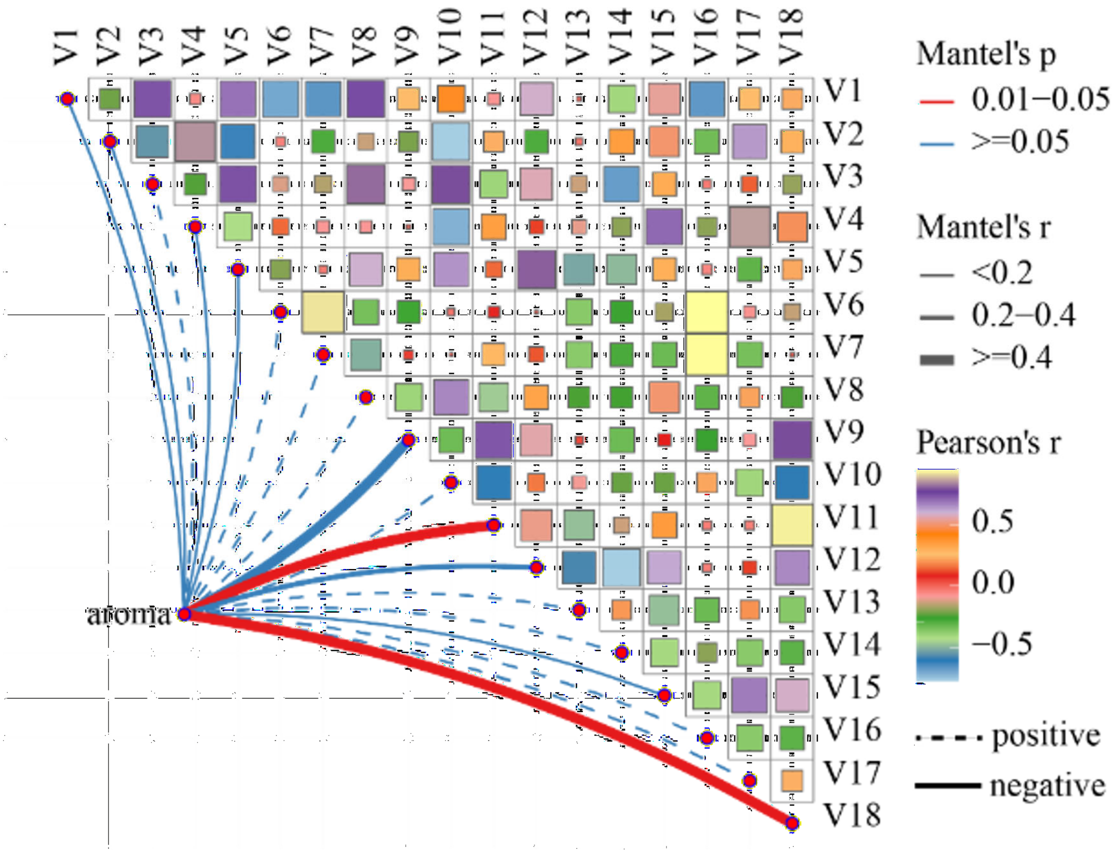

Figure S5. Correlation analysis between tea aroma scores and key volatile compound contents under different treatments. The corresponding volatile compounds for the codes (V1, V2, V3) are listed in Table S4.
